# Hosts and vectors of *Trypanosoma cruzi* discrete typing units in the Chagas disease endemic region of the Paraguayan Chaco

**DOI:** 10.1017/S0031182016002663

**Published:** 2017-02-09

**Authors:** NIDIA ACOSTA, ELSA LÓPEZ, MICHAEL D. LEWIS, MARTIN S. LLEWELLYN, ANA GÓMEZ, FABIOLA ROMÁN, MICHAEL A. MILES, MATTHEW YEO

**Affiliations:** 1 Departamento de Medicina Tropical, Instituto de Investigaciones en Ciencias de la Salud, Universidad Nacional de Asunción – UNA, San Lorenzo CP 2160, Paraguay; 2 Department of Pathogen Molecular Biology, London School of Hygiene and Tropical Medicine, Keppel Street, London WC1E 7HT, UK; 3 Centro para el Desarrollo de la Investigación Científica (CEDIC)/Díaz Gill Medicina Laboratorial/Fundación Moisés Bertoni, Asunción, Paraguay

**Keywords:** *Trypanosoma cruzi*, Paraguayan Chaco, triatomine vectors, armadillos, discrete typing units

## Abstract

Active *Trypanosoma cruzi* transmission persists in the Gran Chaco region, which is considered hyperendemic for Chagas disease. Understanding domestic and sylvatic transmission cycles and therefore the relationship between vectors and mammalian hosts is crucial to designing and implementing improved effective control strategies. Here we describe the species of triatomine vectors and the sylvatic mammal reservoirs of *T. cruzi*, in different localities of the Paraguayan and Bolivian Chaco. We identify the *T. cruzi* genotypes discrete typing units (DTUs) and provide a map of their geographical distribution. A total of 1044 triatomines and 138 sylvatic mammals were captured. Five per cent of the triatomines were microscopically positive for *T. cruzi* (55 *Triatoma infestans* from Paraguay and one sylvatic *Triatoma guasayana* from Bolivia) and 17 animals (12·3%) comprising eight of 28 (28·5%) *Dasypus novemcinctus*, four of 27 (14·8%) *Euphractus sexcinctus*, three of 64 (4·7%) *Chaetophractus* spp. and two of 14 (14·3%) *Didelphis albiventris*. The most common DTU infecting domestic triatomine bugs was TcV (64%), followed by TcVI (28%), TcII (6·5%) and TcIII (1·5%). TcIII was overwhelmingly associated with armadillo species. We confirm the primary role of *T. infestans* in domestic transmission, armadillo species as the principal sylvatic hosts of TcIII, and consider the potential risk of TcIII as an agent of Chagas disease in the Chaco.

## INTRODUCTION

*Trypanosoma cruzi* is the causative agent of Chagas disease, a neglected human protozoan disease that is estimated to affect approximately six million people, spanning 21 endemic Latin American countries, with 60–80 million at risk of infection (WHO, 2015). *Trypanosoma cruzi* is genetically heterogenous, infecting a large number of mammal species and transmitted by haematophagous triatomine insect vectors. Nomenclature is historically complicated, but *T. cruzi* is currently subdivided into six subspecific groups, referred to as genetic lineages or discrete typing units (DTUs) and designated TcI to TcVI (Zingales *et al.* 2012). A cohort of geographically disparate bat trypanosomes, provisionally designated as TcBat, has been shown to share phylogenetically close affiliations with TcI (Marcili *et al.* 2009*a*), although more detailed sampling is required to confirm this as a formal taxonomic group. In a recent review, analysing more than 400 sequences with two mitochondrial (*CytB* and *COII*) and one nuclear gene (*Gpi*), authors propose three significant reliable mitochondrial clades, named mtTcI, mtTcII and mtTcIII, instead of seven (Barnabé *et al*. 2016). Phyloepidemiology and host vector associations of *T. cruzi* are complex, but have been partially resolved (Yeo *et al.* 2005; Miles *et al.* 2009; Messenger *et al.* 2015; Brenière *et al*. 2016). TcI is widespread through the Americas. This is the major DTU found infecting *Didelphis* opossums in nature, which is believed to be its most ancestral host. TcI was reported predominating in domestic transmission cycles in northern countries of South America (for example, Colombia and Venezuela) and in Central America (Miles *et al*. 2009). TcIII and TcIV primarily circulate in sylvatic transmission cycles, the former especially associated with armadillos (Yeo *et al.* 2005) and the latter with a variety of sylvatic mammal species (Miles *et al.* 2009). TcIV is a secondary cause of Chagas disease in Venezuela. TcIII is rarely reported from domestic transmission cycles. In contrast, TcII, TcV and TVI predominate in domestic transmission cycles in Southern Cone countries of South America (Miles *et al.* 2009; Messenger *et al.* 2015; Brenière *et al*. 2016). Remarkably, TcV and TcVI are known to be natural hybrids derived from genetic exchange between TcII and TcIII in recent evolutionary history and are at present strongly associated with domestic transmission cycles (Zingales *et al.* 2012; Brenière *et al*. 2016).

*Trypanosoma cruzi* infection is considered primarily a zoonosis and as such eradication is not possible. Effective control of Chagas disease is achieved by interrupting vectorial transmission, primarily through residual insecticide-spraying to reduce domestic infestation and also by serological surveillance and interruption of transmission by blood transfusion, organ donation and congenitally (WHO, 2015).

There has been remarkable progress in controlling Chagas in some regions of the Americas. However, the Gran Chaco region, which includes territories of Argentina, Bolivia and Paraguay, is currently considered one of the most difficult regions for effective control and remains highly endemic (Hotez, 2014*a*). The land area is vast and arid, with a low population density consisting of small widely dispersed communities of low socioeconomic status (Gürtler, 2009). More than 20 ethnic groups live in marginalized conditions with minimal access to health care provisions (Gracey and King, 2009; Hotez, 2014*b*). Indigenous communities of the Gran Chaco show consistently high seroprevalence of human *T. cruzi* infection, ranging from 12 to 83% with local variation (Canese and Brice, 1977; Rojas de Arias *et al.* 1993; Moretti *et al.* 2010; Samuels *et al.* 2013).

Although the success of vector control interventions in some areas of the Chaco has substantially reduced disease incidence, the main challenge is the long-term sustainability, and in particular, entomological surveillance. Reinfestation of treated dwellings, when the residual effect of insecticides decreases, is a common feature especially in areas with peridomestic vectors and/or reinvasion by secondary vectors from the sylvatic environment (Provecho *et al.* 2014; Gaspe *et al.* 2015). Other obstacles adversely affecting control in the Chaco include the low efficacy of pyrethroid insecticide spraying on often poorly constructed peridomestic structures in this region (Gürtler *et al.* 2007; Cécere *et al.* 2013). Of further concern, is the appearance of *Triatoma infestans* populations resistant to pyrethroid insecticides in localities of northern Argentina and southern Bolivia (Lardeux *et al.* 2010; Gurevitz *et al.* 2012). Sylvatic populations of *T. infestans* have been identified in the Chaco and pose a potential risk of reinvasion (Noireau *et al*. 1997*a*; Ceballos *et al.* 2011; Quisberth *et al*. 2011; Rolón *et al.* 2011) as do secondary vectors, including *Triatoma sordida* (Almeida *et al.* 2000; Damborsky *et al.* 2001; Feliciangeli *et al.* 2003; Lauricella *et al.* 2005); additionally sylvatic mammals are potential reservoirs of infection, and all of these factors may confound effective control. This study ascertains the different triatomine species present in the region, the mammal species infected and the associated circulating *T. cruzi* DTUs. Through a better understanding of the *T. cruzi* transmission dynamics we aim to improve control strategies.

## MATERIALS AND METHODS

### Fieldwork

Fieldwork collections were performed from 2002 to 2008, with the objective of obtaining and genotyping new isolates of *T. cruzi* from triatomine bugs and sylvatic mammals. Isolates obtained in previous surveys (Yeo *et al.* 2005; Llewellyn *et al.* 2009, Rojas de Arias *et al.*, manuscript in preparation) were also included to generate a more detailed picture of the distribution of *T. cruzi* DTUs in the Chaco region.

### Study area

**Fig. 1.**
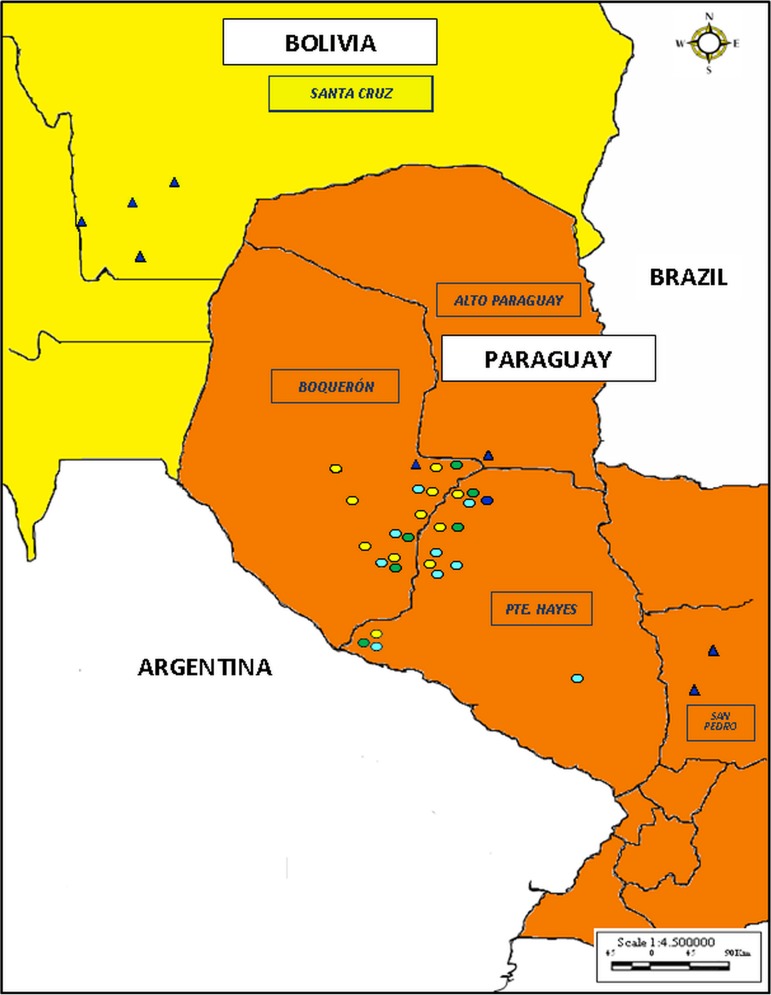
Distribution of *Trypanosoma cruzi* DTUs in the study area. Map illustrating study sites in Paraguay (orange) and Bolivia (yellow) and the distribution of trypanosomes characterized. Circles and triangles represent isolates from domestic and sylvatic cycles, respectively. Colours indicate different *Trypanosoma cruzi* DTUs. Green, TcII; blue, TcIII; yellow, TcV and light blue, TcVI.

The study area encompasses the Paraguayan Chaco (western region), Bolivian Chaco (southern region) and also three further Paraguayan localities 250 km northeast of Asunción (San Pedro, San Alfredo and Aguapey), within the Department of San Pedro. Study areas are shown in Fig. 1. In total, data were acquired from 28 localities, 24 Paraguayan and four Bolivian. Of the 24 Paraguayan localities, 21 were situated in the Paraguayan Chaco spanning three departments (Boquerón, Presidente Hayes and Alto Paraguay). Within the Department of Boquerón 12 localities were studied: Betania, Campo Loro, Campo Nuevo, Campo Salado, Cesarea, Galilea, Jerico, Campo Alegre, Casuarina, Jotoisha, Tiberia and Samaria. Within the Department of Presidente Hayes a further eight localities were included: Cerrito, Estancia Salazar, 12 de Junio, 20 de abril, Campo Largo, 10 Leguas, Fischat and Jope. Lastly, the one locality from the third department was Don Anibal ranch. These aforementioned localities have been under epidemiological surveillance by the National Program Control of Chagas since 2001. Further details regarding localization, ethnic group and estimated population size of these communities is shown in Table A1 (Appendix A). The remaining three localities lie within the Department of San Pedro (Fig. 1), in the Southeast Chaco: San Pedro, San Alfredo and Aguapey. Localities from the Bolivian Chaco region, San Antonio, Mora, Cuatro Cañadas and Gutierrez were all from Santa Cruz Department.

Indigenous communities consist of nomadic hunters, gatherers and fishermen with some groups of sedentary farmers from distinct linguistic groups (Rojas de Arias, 2003). Dwellings are typically of low quality, walls constructed of wattle and brick, adobe or palm trunk, soil floor with straw, and palm leaves or tin as roofing material. Villages are located in peripheral areas surrounding Mennonite towns. Domestic animals consist primarily of dogs and chickens. Further to the Northeast and in the Bolivian Chaco land use is predominantly for cattle farming and agriculture. To the east of the Paraguayan Chaco, the Department of San Pedro is characterized by more abundant vegetation and intensive agricultural land use.

### Collection of triatomine bugs and sylvatic mammals

Each locality was visited only once. Most of the collection of domiciliary and peridomiciliary triatomines was made by manual searches (1·0 person hour per house) (Gürtler *et al.* 1998). Domestic areas inspected included walls, interior side of roofs, furniture and bedding. Peridomestic searches encompassed chicken coups, wood piles and other peridomestic structures (Gurevitz *et al.* 2011). In Campo Loro, Estancia Salazar and Fischat localities domestic bugs were obtained by the collection of local inhabitants. Sylvatic triatomines were collected by the use of live-bait Noireau traps (Noireau *et al*. 2000), manual dissection of natural ecotopes (bird nests, fallen trees and scrub) and light traps (Vazquez-Prokopec *et al.* 2006). Light traps were left overnight and checked in the morning. Captured bugs were placed in labelled containers, the developmental stage noted and identified to the level species by trained personel according to Lent and Wygodzinsky (1979). The captured nymphs were raised in laboratory conditions until they reached the adult stage to confirm the species. Sylvatic mammals were captured by the use of collapsible ‘live-traps’ including Sherman (H.B. Sherman Trap, Inc., Tallahassee, FL) and Tomahawk (Tomahawk Live Trap Co., WI), baited with a mixture of peanut butter, ripe banana and oats. Ten traps per night were set at approximately 10 m intervals on animal trails, near burrow entrances, in dense scrub or close to fallen trees. Traps were left *in situ* for 3 days, set at sunset and examined at dawn, where applicable local hunters were hired to collect live mammals. All specimens captured were sexed, identified to the species level (Neris *et al.* 2002), and released unharmed after processing (see below).

### Isolation and characterization of trypanosomes

Trypanosomes were obtained from animals via xenodiagnosis and from triatomines by haemoculture and xenoculture as previously described (Miles, 1993). Mammals were first anaesthetized by intramuascular injection using ketamine (Holliday-Scott^®^, 50–80 mg kg^−1^ body weight). Animal-handling procedures were in accordance with the American Society of Mammalogists (Sikes and Gannon, 2011). To excluded the presence of mixed infection biological clones of *Trypanosoma cruzi* were obtained by direct culture of infected triatomine feces, onto solid medium agar plates, as described previously by Yeo *et al.* (2007), five clones of each isolate were expanded in culture and DNA extracted [DNeasy kits (QIAGEN™)].

Genetic characterization of DTUs was undertaken using a combination of amplicon profiles from four different polymerase chain reactions (PCR) details of which are shown in Table 1. Genetic targets were the D7 divergent domain of the 24Sα rRNA (Souto *et al.* 1996), the size variable domain of 18S rRNA sequence (Brisse *et al.* 2000), the non-transcribed spacer of the mini-exon gene (Souto *et al.* 1996) and the PCR–restriction fragment length polymorphism (PCR–RFLP) of the intergenic region of the heat-shock protein 60 (*HSP60*) gene (Lewis *et al.* 2009). Primers and reaction conditions are described in Table B1 (Appendix B). A panel of reference strains, encompassing the known DTUs, was obtained from the London School of Hygiene and Tropical Medicine cryobank repository and consisted of X10 Clone I (DTU TcI), Esmeraldo-cl3 (DTU TcII), Arma 13 (DTU TcIII), CAN III (DTU TcIV), SC43 (DTU TcV) and CL Brener (DTU TcVI).

**Table 1.** DTU discrimination based on PCR amplification products (bp)^a^

PCR reaction	TcI	TcII	TcIII	TcIV	TcV	TcVI	DTU identification
24Sα rRNA	110	125	110	120^b^	110/125	125	TcIV, TcV
18S rRNA	160	165	165	155	165	165	TcI, TcIV
Mini-exon	350	300	250^a^ or none^c^	400^a^ or none^d^	300	300	TcI, TcIV, TcIII
RFLP–PCR^e^ (*HSP60*/*Eco*RV)	462	462	314/148	462	462/314/148	462/314/148	TcIII^f^

a According to Yeo *et al.* (2005) and Lewis *et al.* (2009).

b Brisse *et al.* (2000) reported bands of 125 bp in one strain (Saimiri 3) and of 130 bp for three strains of North American origin. Band of 117 bp was reported by Kawashita *et al.* (2001).

c According to Brisse *et al.* (2000). They reported product a low intensity band of 300 bp in two strains (M6241 cl6 and M5631 cl5).

d According to Brisse *et al.* (2000).

e According to Westenberger *et al.* (2005) and Lewis *et al.* (2009).

f Differentiates between TcII and TcVI.

## RESULTS

### Triatomines

A total of 1044 triatomine bugs were included in the current study, 1037 from Paraguay and seven from the Bolivian Chaco. *Triatoma infestans* (*n* = 715) was found in both domestic and peridomestic environments (*n* = 245 in domestic; *n* = 470 in peridomestic) and *T. sordida* (*n* = 203) only in peridomestic environments (Table 2). Adults, fourth- and fifth-instar nymphs were collected from both species in both areas. In Betania, Campo Salado, Galilea both species shared the same niche in chicken coops. In the localities of Campo Nuevo, Cesarea and Samaria only *T. sordida* was present, while in Estancia Salazar only *T. infestans* was found. Although *T. infestans* was collected from both peridomestic and domestic areas, only domestic specimens were microscopically positive for *T. cruzi*. Thus, 55 (5·4%) of *T. infestans* were positive, including adults (*n* = 41), fourth-instar (*n* = 10) and fifth-instar (*n* = 4) nymphs. Positive triatomines were from the localities of Jerico (*n* = 31), Galilea (*n* = 2), Betania (*n* = 1), Campo Loro (*n* = 5), Estancia Salazar (*n* = 8), Jope (*n* = 5) and Fischat (*n* = 3). In the sylvatic area, 115 of adults (*n* = 107) and nymphs (*n* = 8) of *Triatoma guasayana*, three adults of *T. sordida*, and one female of *Triatoma platensis* were captured from the localities of Campo Loro, Don Anibal ranch and Betania respectively and they were microscopically negative. Triatomines obtained from Bolivian Chaco by the cooperation of local inhabitants included seven sylvatic adult bugs, *T. sordida* (one specimen) and *T. guasayana* (six specimens). One *T. guasayana* specimen from the locality of Mora was positive.

**Table 2.** Summary table: species captured, location, ecotopes, number of positives and DTUs observed

Country	Department	Locality	Species	Total captured	Ecotope	Positive	*Trypanosoma cruzi* DTU				
							II	III	V	VI	ND
Paraguay	Boquerón	Betania	*Triatoma infestans*	97	82 peridomestic 15 Domestic	1^a^			1		
			*Triatoma sordida*	18	Peridomestic	–					
			*Triatoma platensis*	1	Sylvatic	–					
		Campo Loro	*Triatoma infestans*	5	Domestic^b^	5	1^c^		5		
			*Triatoma guasayana*	10	Sylvatic	–					
			*Dasypus novemcinctus*	2	Sylvatic	2		5^c^			
			*Euphractus sexcinctus*	18	Sylvatic	1		1			
			*Chaetophractus* spp.	41	Sylvatic	1		1			
		Campo Nuevo	*Triatoma sordida*	39	Peridomestic	–					
		Campo Salado	*Triatoma infestans*	76	Peridomestic	–					
			*Triatoma sordida*	40	Peridomestic	–					
		Cesarea	*Triatoma sordida*	5	Peridomestic	–					
		Galilea	*Triatoma infestans*	188	186 Peridomestic, 2 Domestic	2^a^			2		
			*Triatoma sordida*	69	Peridomestic	–					
		Jerico	*Triatoma infestans*	230	23 Peridomestic, 207 Domestic	31^a^			26	1	4
		Campo Alegre^e^	*Triatoma infestans*	–	Domestic	–			3		
		Casuarina^e^	*Triatoma infestans*	–	Domestic	–	1		18	1	
		Jotoisha^e^	*Triatoma infestans*	–	Domestic	–			8		
		Samaria	*Triatoma sordida*	32	Peridomestic	–					
		Tiberia^c^	*Triatoma infestans*	–	Domestic		1			1	
	Presidente Hayes	Estancia Salazar	*Triatoma infestans*	111	103 Peridomestic, 8 Domestic	8^a^				4	4
		12 de Junio^e^	*Triatoma infestans*	–	Domestic				16	21	
		20 de abril^e^	*Triatoma infestans*	–	Domestic					6	
		Campo Largo^e^	*Triatoma infestans*	–	Domestic		1		6		
		10 Leguas^e^	*Triatoma infestans*	–	Domestic					2	
		Fischat	*Triatoma infestans*	3	Domestic^b^	3	3^c^		1	1	
		Jope	*Triatoma infestans*	5	Domestic	5	2	2	1	1^c^	
		Cerrito	*Dasypus novemcinctus*	2	Sylvatic	–					
			*Euphractus sexcinctus*	1	Sylvatic	–					
			*Chaetophractus* spp.	8	Sylvatic	–					
	Alto Paraguay	Don Anibal ranch	*Triatoma guasayana*	105	Sylvatic	–					
			*Triatoma sordida*	3	Sylvatic	–					
			*Dasypus novemcinctus*	1	Sylvatic	–					
			*Euphractus sexcinctus*	1	Sylvatic	1		1			
			*Chaetophractus* spp.	9	Sylvatic	1		1			
			*Cabassous* spp.	1	Sylvatic	–					
	San Pedro	Colonia San Alfredo	*Dasypus novemcinctus*	8	Sylvatic	1		1			
			*Didelphis albiventris*	2	Sylvatic	1					1
		Aguapey	*Dasypus novemcinctus*	6	Sylvatic	–					
			*Didelphis albiventris*	12	Sylvatic	1					1
		San Pedro^c^	*Dasypus novemcinctus*	–	Sylvatic	–		3			
Bolivia	Santa Cruz	Gutierrez^b^	*Triatoma guasayana*	1	Sylvatic	–					
			*Dasypus novemcinctus*	2	Sylvatic	1		1			
			*Euphractus sexcinctus*	5	Sylvatic	–					
			*Chaetophractus* spp.	3	Sylvatic	1		1			
			*Tamandua tetradactyla*	1	Sylvatic	–					
		San Antonio^b^	*Triatoma guasayana*	2	Sylvatic	–					
			*Dasypus novemcinctus*	1	Sylvatic	–					
			*Chaetophractus* spp.	3	Sylvatic	–					
		Mora^b^	*Triatoma guasayana*	3	Sylvatic	1		1			
			*Triatoma sordida*	1	Sylvatic	–					
			*Dasypus novemcinctus*	6	Sylvatic	4		4			
			*Euphractus sexcinctus*	2	Sylvatic	2		2			
			*Dasyprocta* spp.	2	Sylvatic	–					
			*Nasua nasua*	1	Sylvatic	–					
		Cuatro Cañadas^d^	*Dasypus novemcinctus*	–	Sylvatic	–		6			
			*Euphractus sexcinctus*	–	Sylvatic	–		2			
Total				1182		73	9^c^	32^c^	87	38^c^	10

a, domestic; b, captured by local inhabitants; c, include samples from Yeo *et al.* (2005); d, include samples from Llewellyn *et al.* (2009); e, include samples from Rojas de Arias *et al.* (in preparation); ND, not determined.

### Sylvatic mammals

A total of 138 mammals were included in the current study, 26 from the Bolivian Chaco and 112 from Paraguayan localities. From Bolivia animals representing six species were examined, *Euphractus sexcinctus* (*n* = 7), *Dasypus novemcinctus* (*n* = 9), *Chaetophractus* spp. (*n* = 6), *Tamandua tetradactyla* (anteaters, *n* = 1), *Dasyprocta* spp. (agutí, *n* = 2) and *Nasua nasua* (coati, *n* = 1). Eight animals (30%) were found to be infected by haemoculture and/or xenodiagnosis: five *D. novemcinctus*, two *E. sexcinctus* and one *Chaetophractus* spp. from Gutierrez and Mora localities.

From Paraguayan localities a total of 112 sylvatic animals of five species were captured and examined. In the Chaco localities, 84 mammals included four different armadillo species: *E. sexcinctus* (*n* = 20), *D. novemcinctus* (*n* = 5), *Cabassous* spp. (*n* = 1) and *Chaetophractus* spp. (*n* = 58). Six animals (7%) from Campo Loro and Don Anibal ranch were infected: two *E. sexcinctus*, two *Chaetophractus* spp. and two *D. novemcinctus*. All the specimens captured in Cerrito were negative by microscopy, xenodiagnosis and haemoculture. Twenty-eight animals were captured from San Pedro. They included *D. novemcinctus* (*n* = 14) and *Didelphis albiventris* (opossum, *n* = 14). Three animals (11%) from Colonia San Alfredo and Aguapey, one armadillo and two opossums, were infected.

### Trypanosome isolates

A total of 166 *T. cruzi* isolates were included in this study, consisting of 63 new field isolates and a further 103 obtained from previous collections, as described below. Table 2 summarizes the origins of the isolates.

Sixty-three isolates were genotyped to DTU; ten from eight triatomines and two *Didelphis* spp., could not be maintained in culture and were excluded. The isolates from previous collections (Table 2) included 12 that had been genotyped, six from domestic *T. infestans* and six from sylvatic armadillos from the Chaco region of Paraguay (Yeo *et al.* 2005). Eighty-three isolates originated from domestic *T. infestans* (Rojas de Arias *et al.*, in preparation). Eight isolates were from sylvatic armadillos in Bolivia (Llewellyn *et al.* 2009).

### Characterization of trypanosome isolates

**Fig. 2.**
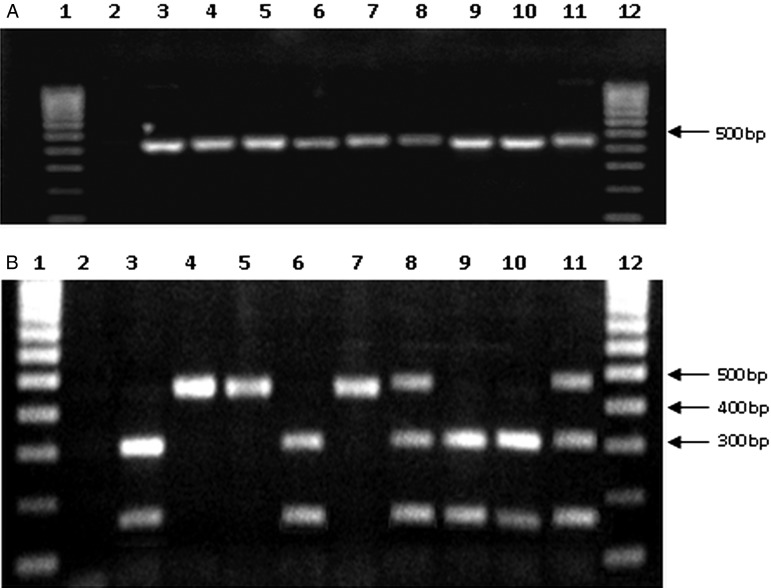
Agarose gel electrophoresis of PCR–RFLP products from *HSP60*/*Eco*RV of selected *Trypanosoma cruzi* isolates. Samples from sylvatic and domestic hosts in Paraguay and Bolivia: A: products without digestion, B: products after of digestion by *Eco*RV. Lanes: 1 and 12 contain hyperladder 4; 2: negative control; 3: TcIII from sylvatic *Triatoma guasayana* in Bolivia; 4–5: TcII from domestic *T. infestans* in Paraguay; 6–7–8: TcIII, TcII, TcVI reference strains, respectively; 9–10: TcIII from domestic *T. infestans* in Paraguay; 11: TcVI from domestic *T. infestans* in Paraguay.

Amplicon sizes obtained with new trypanosome isolates were as expected for the corresponding DTU, according to previous surveys (Yeo *et al.* 2005; Lewis *et al.* 2009). Examples for PCR–RLFP *HSP60*/*Eco*RV that differentiate between TcIII, TcII and TcVI are shown in Fig. 2. Domestic *T. infestans* (*n* = 136) were infected with TcII, TcIII, TcV and TcVI. The most common DTU was TcV (64%), followed by TcVI (28%), TcII (6·6%) and TcIII (1·5%). Twenty-nine sylvatic isolates examined from different armadillo species and one *T. guasayana* (Bolivia) showed an amplicon profile corresponding to DTU TcIII. Five biological clones from each one of the 63 new field isolates were genotyped to detected mixed infections, but none were found.

### Spatial distribution of DTUs

The locality with the most *T. cruzi* diversity was Jope, with all 4 DTUs circulating in the domestic transmission cycle, followed by Jerico, Casuarina and Fischat with three different DTUs. DTU distribution in the study areas is shown in Fig. 1 and Table 2. Within the domestic transmission cycle nine isolates of TcII were identified, all originated from *T. infestans* from six Chaco localities: Campo Loro (*n* = 1), Tiberia (*n* = 1), Casuarina (*n* = 1), Jope (*n* = 2), Campo Largo (*n* = 1) and Fischat (*n* = 3). DTU TcV was the most widely dispersed DTU in domestic areas, observed in 11 localities,which were Campo Loro (*n* = 5), Campo Largo (*n* = 6), Jerico (*n* = 26), 12 de Junio (*n* = 16), Casuarina (*n* = 18), Fischat (*n* = 1), Jope (*n* = 1), Galilea (*n* = 2), Campo Alegre (*n* = 3), Jotoisha (*n* = 8) and Betania (*n* = 1). Lastly, TcVI was identified in domestic areas of nine localities: Jerico (*n* = 1), Casuarina (*n* = 1), Tiberia (*n* = 1), Estancia Salazar (*n* = 4), 12 de Junio (*n* = 21), 20 de abril (*n* = 6), 10 Leguas (*n* = 2), Fischat (*n* = 1) and Jope (*n* = 1).

TcIII was predominant among sylvatic isolates and widespread in different armadillo species, including *D. novemcinctus* (*n* = 20), *E. sexcinctus* (*n* = 6) and *Chaetophractus* spp. (*n* = 3) from both Paraguay and Bolivia. TcIII was rarely observed in the domestic environment, being found in only two *T. infestans* specimens from the Jope locality of the Paraguayan Chaco. A single sylvatic specimen of *T. guasayana* from Mora locality (Bolivia) also harboured TcIII.

## DISCUSSION

### Triatomines and mammal species

#### Triatomines

Four triatomine species were found in the current study: *T. infestans* (*n* = 715), *T. sordida* (*n* = 203), *T. guasayana* (*n* = 115) and *T. platensis* (*n* = 1). *Triatoma infestans* was the only species found in the domestic environment and there was no evidence of domiciliation by *T. guasayana* and *T. sordida*. The latter two species were found in peridomestic and/or sylvatic areas. A single specimen of sylvatic *T. platensis* was captured. This presence of *T. infestans* in human dwellings (245 specimens) and peridomestic habitats (470 specimens) confirms that it is the primary vector of human *T. cruzi* infection in the Paraguayan Chaco (Rojas de Arias *et al.* 1990, 1993, 2011; Zelada *et al.* 1998; Acevedo *et al.* 2002). The overall prevalence of *T. cruzi* infection in captured triatomines was 5·4%, and all those infected in Paraguay were domestic *T. infestans*. One explanation is peridomestic *T. infestans* feeding on avian blood in chicken coops, and birds cannot maintain *T. cruzi* infection (Miles *et al.* 2003). Considering only domestic *T. infestans* the infection rate is 22·4%, representing a substantial risk of transmission to both humans and domestic animals. These findings accord with previous studies in the Paraguayan Chaco, which report human seroprevalence ranging from 12 to 83%, and house infestation rates from 26 to 100% (Canese and Brice, 1977; Chapman *et al.* 1984; Rojas de Arias *et al.* 1993, 2011; Rojas de Arias, 2003). Thus, the Chaco region is the most highly endemic for Chagas disease in Paraguay, primarily among native Amerindians of the low socioeconomic status.

In peridomestic environments, two triatomines species were found (*T. infestans* and *T. sordida*). *Triatoma infestans* in peridomestic areas poses a potential risk of re-infestation of buildings if surveillance measures are not continuous. Although peridomestic areas are also insecticide sprayed as part of the national control programme, the residual effect is lower than in the domicile because of exposure to climatic conditions. As a consequence this makes peridomestic bugs more difficult to control, requiring that spraying and surveillance to be more frequent. *Triatoma sordida* is widely distributed throughout Central Brazil, Eastern and Central Bolivia, the Chaco region of Paraguay and northwestern Argentina where it occurs primarily in the sylvatic environment (Lent and Wygodzinsky, 1979; Diotaiuti *et al.* 1995). In the Bolivian Chaco, two putative cryptic species belonging to *T. sordida* complex, named groups 1 and 2, respectively, were recognized circulating in sympatry, using multilocus enzyme electrophoresis (Noireau *et al*. 1998). This species shows great capacity of adaptation to peridomestic sites, especially in association with chickens (Macchiaverna *et al.* 2015). In the current study, all specimens (*n* = 203) were negative for flagellates probably because their principal food sources are avian. A primary domiciliation by *T. sordida* in the Chaco region was described in localities of Velasco Province, Department of Santa Cruz (Bolivia), where 16·2% of bugs were found infected by *T. cruzi*, although the probability of transmission to humans was considered low (Noireau *et al*. 1997*b*; Brenière *et al*. 1998). Three species of sylvatic triatomine were found: *T. guasayana* (*n* = 115), *T. sordida* (*n* = 3) and *T. platensis* (*n* = 1). Their typical ecotopes were fallen trees and dense shrubs, where they were captured using Noireau traps. *Triatoma guasayana* was an active flyer seeking out potential hosts, with most flight activity occurring just after sunset. Both *T. guasayana* and *T. sordida* have been implicated as sylvatic vectors of *T. cruzi* in parts of the dry Chaco region (Wisnivesky-Colli *et al.* 1997; Vezzani *et al.* 2001). In Paraguayan localities, both species were frequently observed near and around households, especially the adults, which have a great capacity for flight (Yeo *et al.* 2005; Rolón *et al.* 2011). Because of these characteristics and their ability to colonize man made structures, they are candidate as secondary vectors. All our sylvatic *T. guasayana* and *T. sordida* captured in Paraguay were not infected with *T. cruzi*, apart from a single sylvatic Bolivian *T. guasayana.* However, infected *T. guasayana* and *T. sordida* have previously been reported in the Argentinean and Bolivian Chaco (Noireau *et al.* 2000; Bar *et al.* 2002; Lauricella *et al.* 2005; Ceballos *et al.* 2009) with average infection rates of 13·3 and 9·1%, respectively. In the Argentinean Chaco, sylvatic *T. sordida* have been reported with high infection rates (38·5%; Bar and Wisnivesky-Colli, 2001; Bar *et al.* 2002).

Here we did not find *T. infestans* in the sylvatic ecotope. However, sylvatic ‘dark morph’ *T. infestans* have been reported in the Chaco region of Bolivia, in nests of *Myiopsitta monachus* (parrot), in bromeliads and hollows of live trees in several localities (Noireau *et al*. 1997*a*, *b*; Brenière *et al.* 2012; Waleckx *et al*. 2012), in the Argentinean Chaco (Ceballos *et al.* 2009, 2011) and Chile (Bacigalupo *et al.* 2006). Prevalence of *T. cruzi* infection is markedly lower in such ‘dark morph’ forms from the Chaco region with prevalence of between 2·5 and 12·5% (Noireau *et al.* 2000; Brenière *et al.* 2012; Waleckx *et al*. 2012) or zero (Ceballos *et al.* 2009, 2011) probably due that avian blood is the more often source of food. A few surveys have previously reported putative sylvatic populations of *T. infestans* in the Paraguayan Chaco, although they were also presumed attributable to dispersed peridomiciliary populations (Velázquez and González, 1959; Usinger *et al.* 1966; Yeo *et al.* 2005). More recently putative sylvatic colonies were discovered using a trained dog (Rolón *et al.* 2011), and these bugs were found 3 km from infested houses. It is significant that this species is capable of surviving in sylvatic ecotopes in at least in some regions of the Paraguayan Chaco. Further research is needed to establish the risk of reinvasion from such sylvatic populations of *T. infestans*.

#### Mammals

Eight species of mammals belonging to five different orders were captured in the study area. The overall prevalence of infection by *T. cruzi* was 12·3% (17/138), although this percentage varied according to the genus. Armadillos were the most common species captured in both regions of Paraguay (Chaco and San Pedro Departments) and from different localities in Bolivia. In two recent surveys performed in the humid Argentinean Chaco, marsupials and rodents together with armadillos were the most frequently captured species (Alvarado-Otegui *et al*. 2012; Orozco *et al*. 2013). The scarce number of marsupials captured and none for rodents in our study is probably related to the environment, since most of our successful collections were from the dry zone of the Chaco. The highest rate of infection was observed in *D. novemcinctus* (28·5%) followed by *E. sexcinctus* (14·8%) and *Chaetophractus* spp. (4·7%). In previous surveys in the same area, infection in armadillos ranged from 3 to 63%, with the highest prevalence in the *Dasypus* (Yeo *et al.* 2005; Llewellyn *et al.* 2009). *Dasypus novemcinctus* and the other armadillo especies are commonly hunted by the inhabitants of rural communities for food or for handicraft products, and they may be kept alive for several days before being used. Thus, infected armadillos pose a risk for bringing sylvatic *T. cruzi* into the domestic habitat. The triatomine vectors involved in sylvatic transmission cycles in the Chaco region remain uncertain. Members of the genus *Panstrongylus* were reported associated with armadillo burrows in Brazil (Grisard *et al.* 2000), Venezuela (Llewellyn *et al.* 2009) and Argentina (Alvarado-Otegui *et al.* 2012). Our finding of one infected sylvatic *T. guasayana* in fallen trees in Bolivia could suggest some role in sylvatic transmission. The omnivorous behaviour of some mammal species also may contribute to their acquisition of infection (Rabinovich *et al.* 2001). The prevalence of *T. cruzi* infection in *Chaetophractus* spp. was lower than the other armadillos. Although this species construct their own burrows, they are nomadic and rarely use the same burrow twice, and thus unlikely to become infested with triatomines. Three other mammal species: *T. tetradactyla* (anteater), *Dasyprocta* spp. (agutí) and *N. nasua* (coati) from Bolivian localities were not infected with *T. cruzi*. Natural infection of *T. tetradactyla* by *T. cruzi* has been reported in Brazil (Miles *et al.* 1981; Bento *et al.* 1992; Fernandes *et al.* 1999) and Colombia (Ramírez *et al.* 2011). In addition, anteaters and coati are the known hosts of *T. rangeli* (Miles *et al.* 1983; Dereure *et al.* 2001).

Two *D. albiventris* of 14 captured (14·2%) from the Department of San Pedro were infected with *T. cruzi*, although isolates were not genotyped. These marsupials are usually found in humid areas, so the dry expanse of some Chaco zones may not be suitable for them. They are frequently observed in close proximity to human populations, and high *T. cruzi* infection rates have been found in Brazil (21·9 and 45·2% prevalence; Grisard *et al.* 2000) and in the humid Chaco of Argentina (36 and 38% prevalence; Alvarado-Otegui *et al.* 2012; Orozco *et al.* 2013). In San Pedro Department, *T. cruzi* has also been found in the terrestrial opossum *Monodelphis domestica* (Yeo *et al.* 2005). Further studies are needed to understand fully the role of marsupials in transmission of *T. cruzi* in Paraguay.

#### Host–vector of *T. cruzi* genotypes in the Paraguayan Chaco

Ours is the most comprehensive survey of *T. cruzi* genotypes in the Paraguayan Chaco region, providing new insight into the transmission dynamics and dispersion among domestic and sylvatic cycles.

TcII, TcIII, TcV and TcVI were circulating in the region, with the hybrids TcV and TcVI being most frequently found, supporting earlier observations (Yeo *et al.* 2005; Lauthier *et al.* 2012; Maffey *et al.* 2012; Pérez *et al.* 2013). TcV and TcVI were predominant and the most dispersed, and found solely infecting *T. infestan*s in the domestic cycle, also in agreement with previous surveys (Chapman *et al.* 1984; Acosta *et al.* 2001; Yeo *et al.* 2005). TcV and TcVI were reported in domestic *T. infestans* in the Bolivian Chaco (Pérez *et al.* 2013), in domestic and peridomestic triatomines (*T. infestans* and *T. sordida*) and domestic dogs and cats in the Argentinean Chaco (Maffey *et al.* 2012; Enriquez *et al.* 2013). Thus, TcV and TcVI constitute the largest current threat to human health, and have been associated with severe chronic manifestations of Chagas disease in the southern Cone countries (Corrales *et al.* 2009; Cura *et al*. 2012; Vicco *et al.* 2012; Lucero *et al.* 2016). TcV and TcVI are infrequently reported in sylvatic cycles: TcV has been observed in one sylvatic *D. novemcinctus* and one *E. sexcinctus* in Paraguay (Yeo *et al.* 2005), in a rodent (*Octodontomys* spp.), three opossums, two ferrets and one skunk in Argentina (de Luca D'oro *et al.* 1993; Montamat *et al.* 1992), and in two sylvatic triatomines (*Triatoma* spp.) from the Bolivian Chaco (M. Llewellyn, unpublished data). There is one record of TcVI in a *D. marsupialis* in the Northeast La Paz (the Jungas and Alto Beni regions) in Bolivia (Valette *et al*. 1988). It has been suggested that the domestic predominance of TcV and TcVI may be due to their recent anthropogenic origin and rapid clonal dissemination with *T. infestans* and human migration (Lewis *et al.* 2011). The occurrence of sylvatic TcV and TcVI in other regions, such as the Atlantic forest, remains to be fully explored.

TcII was found only in domestic *T. infestans*, in agreement with previous surveys in the Paraguayan Chaco, where it is also associated within human infections (Acosta *et al.* 2001; Yeo *et al.* 2005), although in lower frequency than the TcV and TcVI hybrids. TcII has been detected in single triatomines carrying mixed infection with TcVI (Yeo *et al.* 2007), and the presence of TcII may have been underestimated as discriminatory markers have not been applied. Like TcV and TcVI, TcII rarely been reported in sylvatic cycles, although this may reflect limited research. Recently, TcII was reported infecting one sylvatic *T. infestans* in the Bolivian Chaco (Waleckx *et al*. 2012). Likewise, this DTU has been reported from one monkey (Acosta *et al*. 2016) and one *E. sexcinctus*, in Paraguay (Yeo *et al.* 2005) and from sylvatic mammals in Brazil (Fernandes *et al.* 1999; Bhattacharyya *et al.* 2015; Lisboa *et al*. 2015). Sylvatic TcII reservoirs are of particular interest as it is considered to be ancient (Westenberger *et al.* 2005; de Freitas *et al.* 2006).

A striking predominance of TcIII was apparent in sylvatic isolates. Twenty-nine sylvatic armadillos from Paraguay (both regions) and Bolivia, one sylvatic *T. guasayana* (from Bolivia) and two domestic *T. infestans* (from Paraguay) harboured TcIII. TcIII is frequently and widely found in sylvatic habitats with armadillos, particularly the genus *Dasypus* (Yeo *et al.* 2005; Llewellyn *et al.* 2009; Morocoima *et al.* 2012). Armadillos infected with TcIII were also reported in Colombia (Saravia *et al.* 1987), Venezuela (Llewellyn *et al.* 2009; Morocoima *et al.* 2012), Bolivia (Llewellyn *et al.* 2009), Brazil (Lisboa *et al.* 2009; Marcili *et al.* 2009*b*) and Argentina (Alvarado-Otegui *et al.* 2012; Orozco *et al.* 2013). In San Pedro (Paraguay), armadillos and one specimen of *M. domestica* (opossum) were infected previously with TcIII (Yeo *et al.* 2005). One sylvatic *T. guasayana* from the Bolivian Chaco carried TcIII, presumably acquired by feeding on an armadillo; this is the first report of TcIII in *T. guasayana* in Bolivia. *Triatoma guasayana* is frequently found near houses, attracted by light and CO_2_, may therefore introduce TcIII into the domestic cycle. This DTU has also been isolated from terrestrial sylvatic triatomines collected, such as *P. geniculatus, T. rubrovaria, T. brasiliensis* and *T. vitticeps* in Brazil (Póvoa *et al.* 1984; Martins *et al.* 2008; Santos-Mallet *et al.* 2008; Lisboa *et al.* 2009) and from *Panstrongylus* spp. associated with a burrow of *D. novemcinctus* in Venezuela (Llewellyn *et al.* 2009).

Two domestic *T. infestans* from the Chaco region of Paraguay harboured TcIII. Previously in the same region TcIII isolates were obtained from domestic dogs (Chapman *et al.* 1984) and from sylvatic armadillos (Yeo *et al.* 2005), suggesting leaky separation between domestic and sylvatic cycles. Dogs are commonly used for hunting of armadillos in the Chaco region, and dogs may thus introduce TcIII into the domestic transmission cycles, but TcIII has so far not been isolated from human cases of Chagas disease in the Chaco region. In contrast, in the eastern region of Paraguay (Cordillera and Paraguarí Departments), using amplification products of the 24Sα rRNA, mini-exon and hybridization, TcIII was reported in human cases and domestic *T. infestans* (del Puerto *et al.* 2010) as well as from domestic and peridomestic *T. sordida* from Concepción Department (Sánchez *et al*. 2012); this interesting and surprising finding merits follow-up analyses.

Notably, TcI was absent from this survey. TcI has predominantly been found associated with arboreal marsupials, especially the *Didelphis* opossum throughout the Americas but also with rodents and other sylvatic mammals (Yeo *et al.* 2005; Messenger *et al*. 2015). Records of the presence and distribution of TcI in Paraguay are scarce. It has been identified in the direct analysis of feces of domestic *T. infestans* from the Chaco and eastern region (Cura *et al.* 2010), in samples from domestic *T. sordida* in Concepción Department (Sánchez *et al*. 2012), and in one human case from the Chaco region in a mixed infection with TcII (Risso *et al.* 2011). Unfortunately, isolates were not obtained from the two infected opossums from San Pedro Department. TcIV was not found among our many Chaco region isolates, but it has been reported by direct analysis of the intestinal contents of domestic and peridomestic *T. sordida* captured in Concepción Department (eastern region) (Sánchez *et al*. 2012), although additional analyses are required to confirm this observation.

Biological clones analysed in this study produced similar profiles to the original isolates with the combination of PCR techniques used in this study. The cloning technique on solid media has proven to be useful for discriminating mixed infections in *T. cruzi* reservoirs (Yeo *et al.* 2007), especially when a variety of DTUs are circulating sympatrically in the same area.

In summary, the distribution and the high prevalence of TcII, TcV and TcVI in domestic transmission cycles shows the remarkable diversity of *T. cruzi* in the Chaco region of Paraguay. In eight localities more than one *T. cruzi* DTU was present in the domestic transmission cycle showing the great capacity of *T. infestans* in indigenous communities to harbour a variety of *T. cruzi* populations. Furthermore, there is increasing evidence of interaction between domestic and sylvatic transmission cycles. Especially, TcIII in the Jope locality was found in both transmission cycles, suggesting introduction of TcIII into the domestic cycle. TcIII is known to be highly virulent in mice (Morocoima *et al.* 2012) and may therefore prove to be an agent of severe human Chagas disease. The abundance and aggressive nature of *T. guasayana* also carries a risk, if it should adapt to colonization of human dwellings.

The data generated here provide a regional baseline for future research and an indication of potential risks for human health. High-resolution analyses, including comparative genomics, will give further insight into *T. cruzi* transmission dynamics, interactions between sylvatic and transmission and molecular genetics, to inform the much needed improved control of Chagas disease in the Gran Chaco region.

## APPENDIX A

**Table A1.** Localities surveyed in the Chaco (Paraguay and Bolivia) and San Pedro Departments

Country	Department	Locality	Ethnic group	Latitude	Longitude	Inhabitants (approx.)^a^
Paraguay	Boquerón	Betania	Nivaclé	22°36′1·6″S	59°48′54·05″W	363
	Boquerón	Jerico	Nivaclé	22°35′52·71″S	59°48′34·24″W	153
	Boquerón	Cesarea	Nivaclé	22°35′32·59″S	59°49′11·72″W	144
	Boquerón	Samaria	Nivaclé	22°35′55·58″S	59°49′54·41″W	164
	Boquerón	Tiberia	Nivaclé	22°36′41″S	59°50′44·93″W	220
	Boquerón	Galilea	Nivaclé	22°35′4·44″S	59°56′46·44″W	120
	Boquerón	Campo Nuevo	Nivaclé	22°34′26·13″S	59°55′34·76″W	187
	Boquerón	Campo Salado	Nivaclé	22°34′54·84″S	59°57′00·96″W	93
	Boquerón	Campo Alegre	Lengua	22°51′09″S	60°02′10″W	348
	Boquerón	Casuarina	Nivaclé	22°54′21·63″S	60°00′4·45″W	274
	Boquerón	Jotoisha	Nivaclé	22°26′48·86″S	60°37′11·63″W	282
	Boquerón	Campo Loro	Ayoreo	22°4′48·58″S	59°50′29·19″W	651
	Presidente Hayes	12 de Junio	Angaité	22°56′10″S	59°53′45·4″W	290
	Presidente Hayes	20 de abril	Nivaclé	22°57′57·25″S	59°52′1·1″W	84
	Presidente Hayes	Campo Largo	Nivaclé	22°49′44″S	59°54′9·3″W	506
	Presidente Hayes	10 Leguas	Angaité	22°52′8·6″S	59°52′30·3″W	278
	Presidente Hayes	Jope	Nivaclé	22°35′55·91″S	59°47′13·03″W	351
	Presidente Hayes	Fischat	Nivaclé	23°47′27·64″S	60°47′0·09″W	731
	Presidente Hayes	Estancia Salazar	Sanapana	23°4′20·86″S	59°14′12·09″W	472
	San Pedro	San Pedro		24°11′37·59″S	56°34′45·12″W	
	San Pedro	San Alfredo		24°34′11·94″S	56°44′3·52″W	
	San Pedro	Aguapey		24°31′26·82″S	56°47′9·2″W	
Bolivia	Santa Cruz	San Antonio		20°1′1·69″S	63°10′46·32″W	
	Santa Cruz	Mora		18°27′25·75″S	63°12′29·47″W	
	Santa Cruz	Cuatro Cañadas		17°30′58·302″S	61°35′58·80″W	
	Santa Cruz	Gutierrez		19°26′10·63″S	63°31′43·65″W	

Data from indigenous communities include: location, ethnic group and estimated population.

a Atlas de comunidades indígenas del Paraguay, http://www.dgeec.gov.py (2012).

## APPENDIX B

**Table B1.** Primers used and reaction conditions for each one of the PCR reactions performed

PCR reaction	Primers sequence (5′–3)	Reaction mix (20 *µ*L, total volume)							Reaction conditions	Electrophoretic conditions^b^	Restriction digestion reaction
		ddH_2_0	NH_4_ buffer (10×)	MgCl_2_ (50 mm)	dNTP (2 mm)	Primer (20 pm mL^−1^)	DNA target	*Taq*polymerase^a^			
24Sα rRNA	D71 AAG GTG CGT CGA CAG TGT GG D72 TTT TCA GAA TGGCCG AAC AGT	12·2	2	0·6	2	1 each one	1	0·2	30 cycles: 1 min at 94 °C, 1 min at 60 °C, 1 min at 72 °C 1 cycle: 5 min at 72 °C	80 V 0·5× TBE buffer 3% agarose 2 h Hyperladder 5 (Bioline)	
18S rRNA	V1 CAA GCGGCT GGG TGG TTA TTC CA V2 TTG AGG GAA GGC ATG ACA CAT GT	12·2	2	0·6	2	1 each one	1	0·2	Idem to 24Sα rRNA	Idem to 24Sα rRNA	
Mini-exon	TC CCC CCC TCC CAG GCC ACA CTG TC1 GTG TCC GCCACC TCC TTC GGG CC TC2 CCT GCA GGC ACA CGT GTG TGT G	11·2	2	0·6	2	1 each one	1	0·2	27 cycles: 30 s at 94 °C, 30 s at 55 °C, 30 s at 72 °C 1 cycle: 5 min at 72 °C	90 V 0·5× TBE buffer 1·5% agarose 90 min Hyperladder 4 (Bioline)	
PCR–RFLP of *HSP60*	FWD GTG GTA TGG GTG ACA TGT AC REV CGA GCA GCA GAG CGA AAC AT	8	2	2	4	1 each one	1	0·2	30 cycles: 2 min at 94 °C, 30 s at 94 °C, 30 s at 60 °C, 1 min at 72 °C 1 cycle: 10 min at 72 °C	90 V 0·5× TBE buffer 1·5% agarose 1 h Hyperladder 4 (Bioline)	10 *µ*L PCR product 2 *µ*L 10× buffer 0·2 *µ*L BSA^c^ 0·5 *µ*L *Eco*RV 7·3 *µ*L H_2_0 37 °C for 4 h

a Bioline Ltd. London, UK.

b Stained with ethidium bromide and visualized under ultraviolet light.

c Bovine serum albumin acetylated 100×
